# Supplementary figures and images for: Ribosome reinitiation can explain length-dependent translation of messenger RNA
Source: PLoS Comput Biol. 2017 Jun 9;13(6):e1005592. doi: 10.1371/journal.pcbi.1005592 (PMC5482490; doi:10.1371/journal.pcbi.1005592)

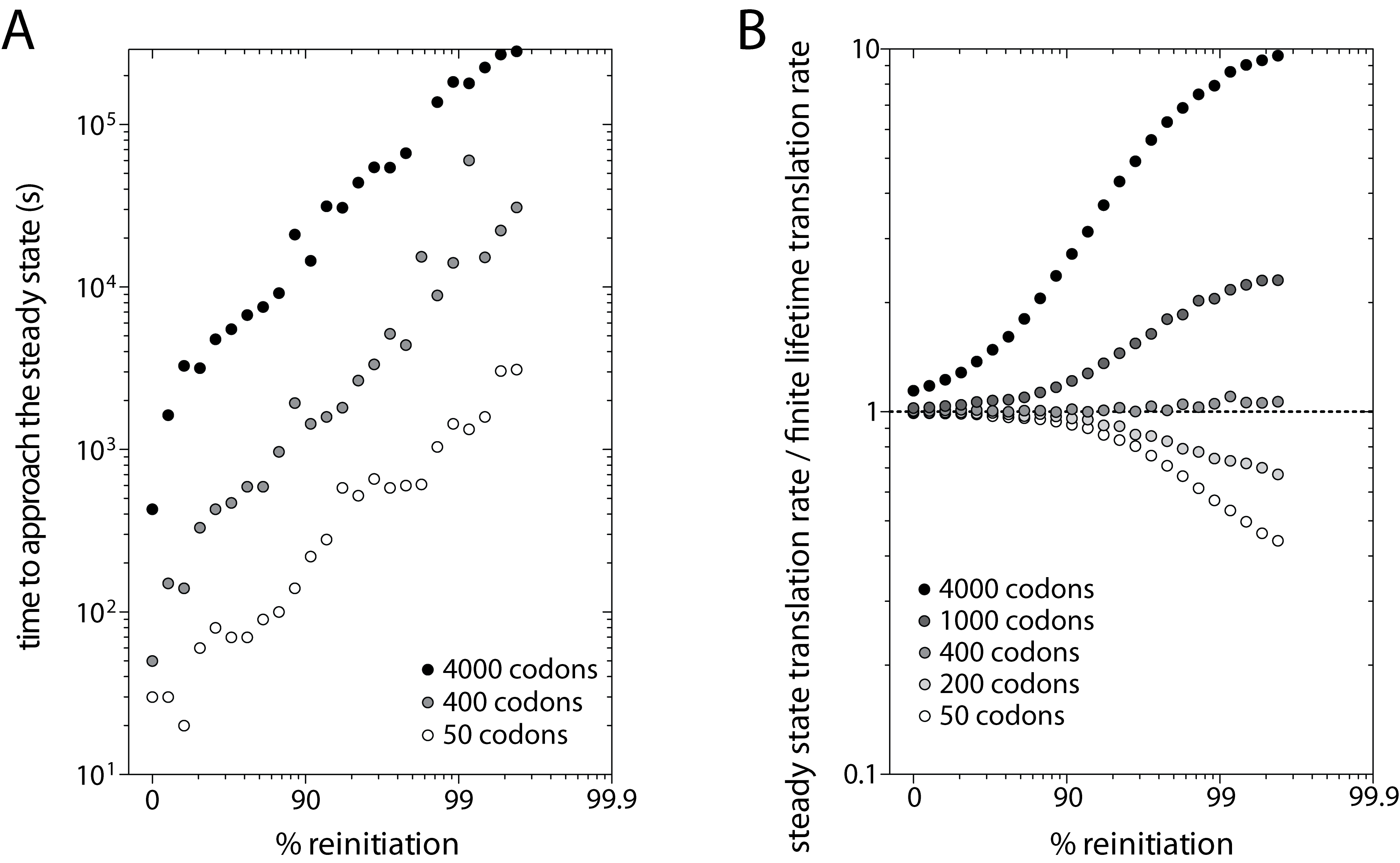

Supplement: S1 Fig — (A) Time to approach the steady state ribosome density at different reinitiation levels for transcripts of different lengths. Simulations were run for 3x105 s using the parameter values described for our general model. De novo initiation rates were adjusted at each reinitiation level so that a 400-codon transcript carried an average of 6 ribosomes at the steady state. The steady state ribosome density was calculated as the average density over the final 104 s of 1000 iterations (if this value showed no directional change over time). The first passage time represents the earliest time point that the average density of 1000 iterations equaled or exceeded the steady state density. Long transcripts (4000 codons) failed to reach the steady state during the 3x105 s run time at reinitiation levels above 99.6%. Data points at higher reinitiation levels were therefore excluded. (B) Steady state translation rate (yield s-1) relative to the average translation rate (yield s-1) on equivalent transcripts with a finite 3000 s lifetime. Deviations from a ratio of 1 represent the magnitude of the misestimation of the translation rate caused by assuming a perpetual steady state on transcripts with finite lifetimes. De novo initiation rates were adjusted at each reinitiation level such that the average 400 codon-transcript carried 6 ribosomes either at the steady state or on average over its 3000 s lifetime. Consequently, all 400 codon transcripts have the same average ribosome density allowing fair comparison. At high reinitiation levels, the steady state approximation overestimates ribosome density on long transcripts and underestimates ribosome density on short transcripts. (TIF) [file pcbi.1005592.s001.tif]

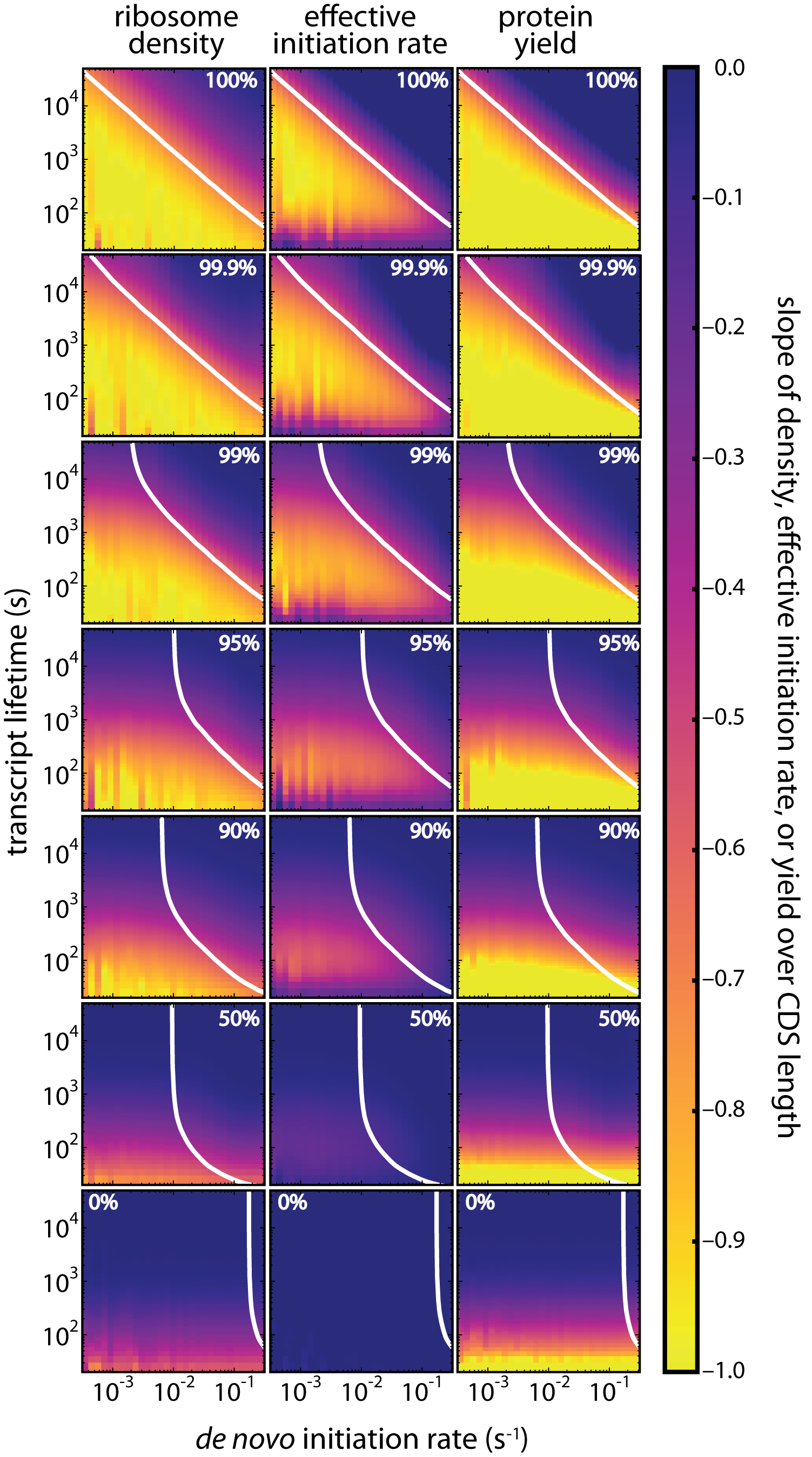

Supplement: S2 Fig — We simulated translation for a wide range of transcript lifetimes and de novo initiation rates. For any given combination of transcript lifetime and de novo initiation rate, we simulated translation for transcripts with different CDS lengths and then calculated the slope of ribosome density, effective initiation rate, and protein yield over CDS length. Slopes are indicated in different colours (see colour bar), reflecting the degree of length-dependence. The white line in each panel shows the de novo initiation rate required at each lifetime such that a 400-codon long transcript carries 6 ribosomes. Our model predicts that high levels of reinitiation can cause length-dependent translation across a wide range of transcript lifetimes and de novo initiation rates. At high levels of reinitiation, length-dependence is strongest for short transcript lifetimes and low de novo initiation rates; since ribosomes are unlikely to leave the transcript, at long lifetimes or high de novo initiation rates, all transcripts eventually become saturated with ribosomes. As a result, the number of ribosomes per transcript becomes proportional to CDS length (since longer transcripts can carry more ribosomes) and ribosome density, the effective initiation rate, and protein yield become similar for all CDS lengths. As the reinitiation level falls, the effective initiation rate becomes dominated by the de novo initiation rate, which is the same for all CDS lengths, and ribosome density, the effective initiation rate, and protein yield all become independent of CDS length. In a linear model of translation (0% reinitiation), negative slopes for density and yield are only seen for extremely short transcript lifetimes simply because the first initiating ribosome fails to reach the stop codon on long transcripts before the maximum lifetime is reached. This effect disappears when transcript lifetimes exceed the time required for the first ribosome to reach the stop codon of the longest transcript [file pcbi.1005592.s002.tif]

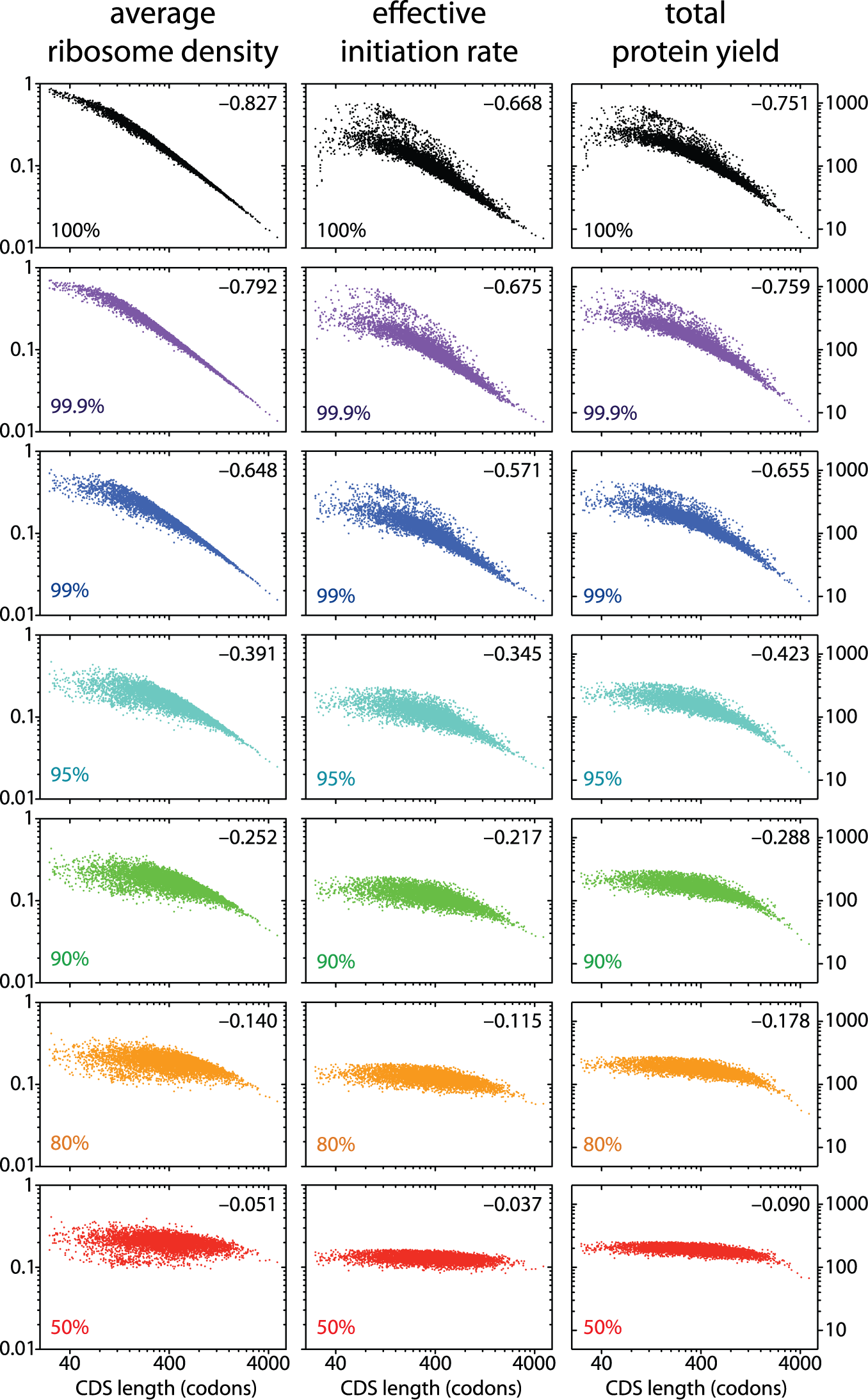

Supplement: S3 Fig — Predicted ribosome density, effective initiation rate, and protein yield for all 5888 budding yeast transcripts simulated at different reinitiation levels using codon-specific decoding rates from [45]. The left y-axis scale applies to ribosome density (left) and effective initiation rates (center); the right y-axis scale applies to protein yield (right). Slopes are indicated in the top-right corner. As in Fig 6, all transcripts had a fixed lifetime of 1553 seconds. De novo initiation rates were adjusted at each reinitiation level so that a 400-codon long transcript with a fixed decoding rate of 10s-1 carried an average of 6 ribosomes. Pearson correlation coefficients between simulated results and empirical measures for each reinitiation level are: (i) ribosome density (n = 5542): 100% = 0.935, 99.9% = 0.932, 99% = 0.908, 95% = 0.784, 90% = 0.619, 80% = 0.377, 50% = 0.083; (ii) effective initiation rate (n = 5348): 100% = 0.742, 99.9% = 0.742, 99% = 0.729, 95% = 0.671, 90% = 0.591, 80% = 0.468, 50% = 0.289; (iii) protein yield (n = 4933): 100% = 0.480, 99.9% = 0.478, 99% = 0.475, 95% = 0.458, 90% = 0.449, 80% = 0.442, 50% = 0.438. Empirical data sources as in Fig 2. (TIF) [file pcbi.1005592.s003.tif]

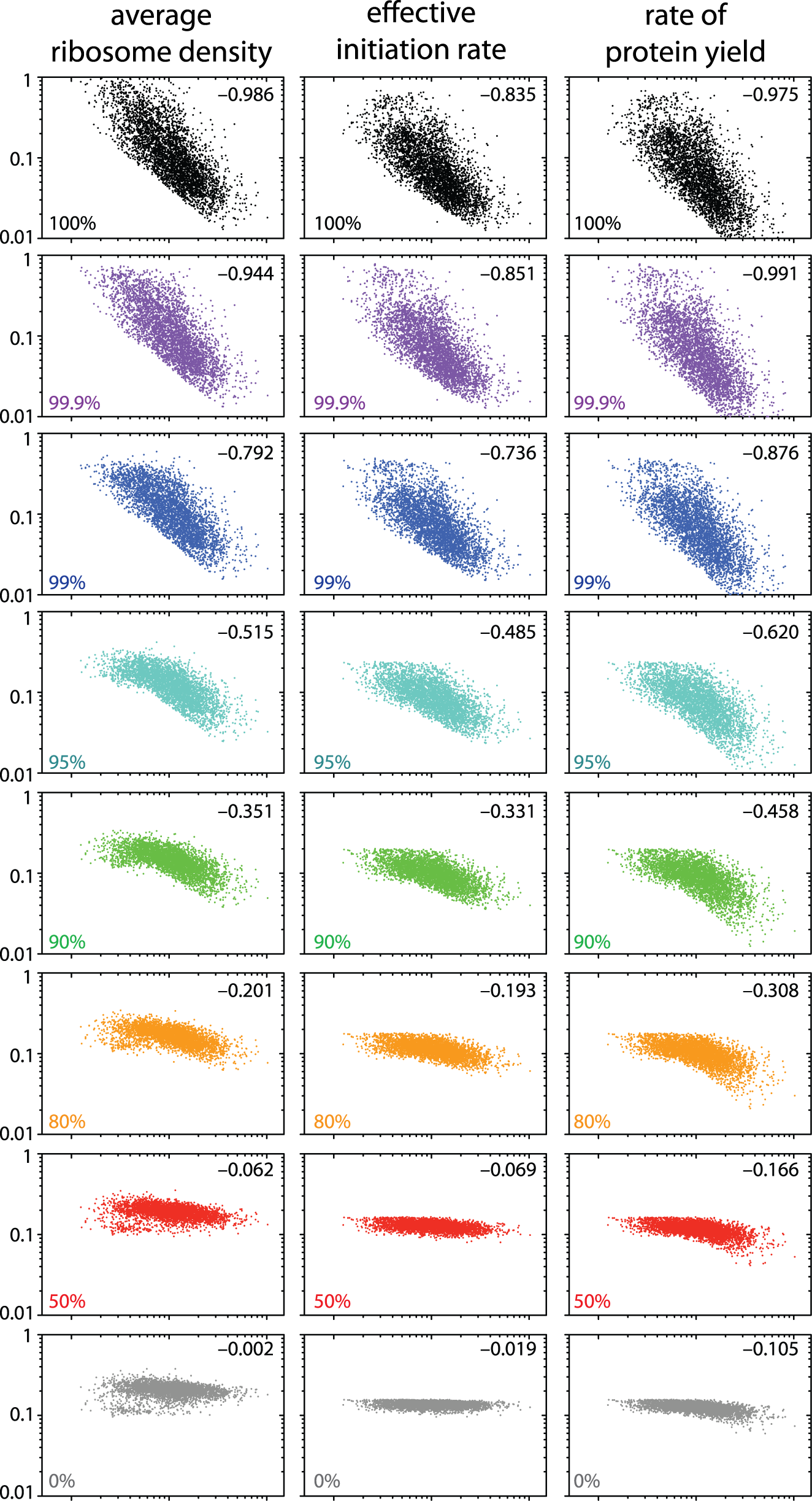

Supplement: S4 Fig — The simulations shown in S3 Fig were repeated using empirical estimates of transcript lifetimes. Transcript lifetimes were calculated using relative abundances from [68] (measured using single-molecule sequencing digital gene expression which eliminates the length-bias associated with RNA-Seq), a total of 36,000 transcripts per cell [69], and nascent transcription rates from [39]. For each transcript, absolute abundance was divided by the transcription rate to obtain the average lifetime. Transcripts with average lifetimes below 400s were excluded to prevent bias towards increased length-dependence (see S2 Fig). Pearson correlation coefficients between simulated results and empirical measures for each reinitiation level are: (i) ribosome density (n = 3829): 100% = 0.767, 99.9% = 0.771, 99% = 0.773, 95% = 0.741, 90% = 0.673, 80% = 0.512, 50% = 0.151, 0% = -0.072; (ii) effective initiation rate (n = 3752): 100% = 0.668, 99.9% = 0.668, 99% = 0.668, 95% = 0.654, 90% = 0.627, 80% = 0.567, 50% = 0.394, 0% = 0.182; (iii) protein yield (n = 3451): 100% = 0.615, 99.9% = 0.620, 99% = 0.609, 95% = 0.578, 90% = 0.561, 80% = 0.546, 50% = 0.535, 0% = 0.527. Empirical data sources as in Fig 2. (TIF) [file pcbi.1005592.s004.tif]

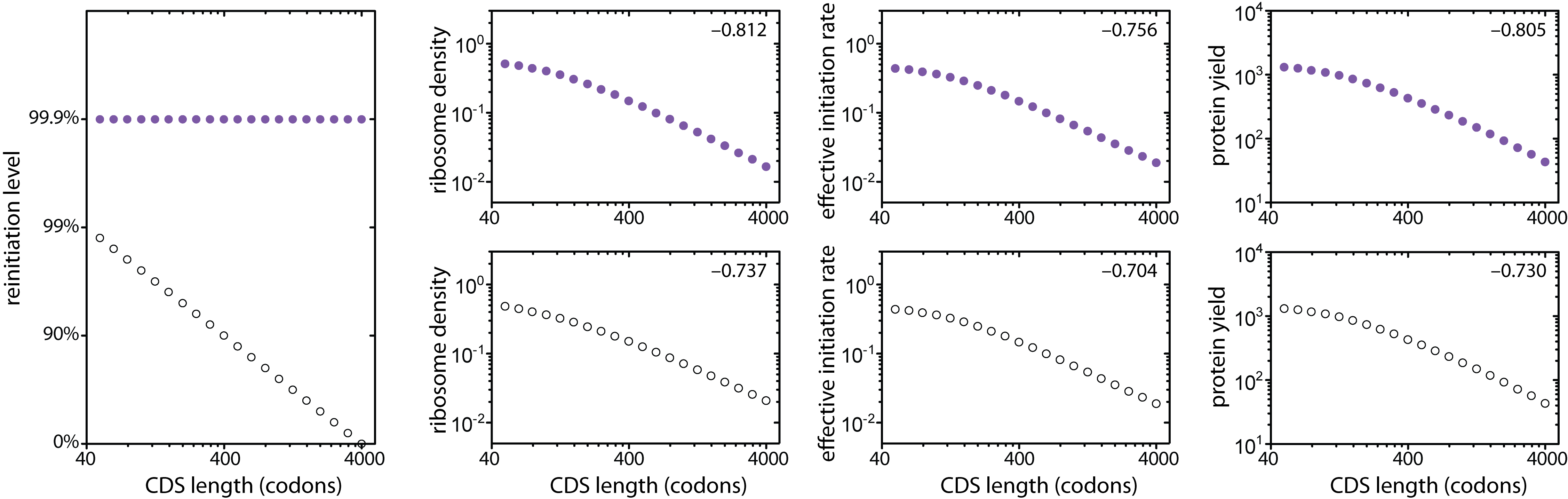

Supplement: S5 Fig — Decreasing reinitiation levels with transcript length (open circles) produces very similar relationships between CDS length and ribosome density, effective initiation rate, and protein yield as does a fixed reinitiation level of 99.9% (purple circles). Slopes of each relationship are shown in the upper right corner of each panel. Length-dependent reinitiation levels capture length dependent translation at much lower reinitiation levels than that required using a fixed reinitiation level. We have arbitrarily chosen to decrease the reinitiation level as a function of CDS length according to the formula 100%*(1- CDS length/4000). The de novo initiation rate was set such that a 400-codon transcript carried an average of 6 ribosomes (99.9% reinitiation = 0.00458s-1, length-dependent reinitiation = 0.02285s-1). (TIF) [file pcbi.1005592.s005.tif]
