# Supplementary material for: Ribosome reinitiation can explain length-dependent translation of messenger RNA
Source: PLoS Comput Biol. 2017 Jun 9;13(6):e1005592. doi: 10.1371/journal.pcbi.1005592 (PMC5482490; doi:10.1371/journal.pcbi.1005592)
Supplement: S1 Table — (DOCX) [file pcbi.1005592.s006.docx]

| Amino acid | Codon | Decoding rate | Amino acid | Codon | Decoding rate | Amino acid | Codon | Decoding rate |
| --- | --- | --- | --- | --- | --- | --- | --- | --- |
| ALA | GCU | 18.2 | GLY | GGC | 27.0 | PRO | CCA | 15.6 |
| ALA | GCC | 11.7 | GLY | GGA | 4.69 | PRO | CCG | 15.6 |
| ALA | GCA | 7.82 | GLY | GGG | 3.13 | SER | UCU | 22.4 |
| ALA | GCG | 7.82 | HIS | CAU | 8.43 | SER | UCC | 14.4 |
| ARG | CGU | 4.21 | HIS | CAC | 13.8 | SER | UCA | 7.28 |
| ARG | CGC | 2.70 | ILE | AUU | 20.3 | SER | UCG | 1.56 |
| ARG | CGA | 2.70 | ILE | AUC | 13.0 | SER | AGU | 3.82 |
| ARG | CGG | 1.56 | ILE | AUA | 3.13 | SER | AGC | 6.26 |
| ARG | AGA | 17.2 | LEU | UUA | 10.9 | THR | ACU | 17.4 |
| ARG | AGG | 1.72 | LEU | UUG | 19.2 | THR | ACC | 11.2 |
| ASN | AAU | 9.56 | LEU | CUU | 0.956 | THR | ACA | 6.26 |
| ASN | AAC | 15.6 | LEU | CUC | 1.56 | THR | ACG | 1.56 |
| ASP | GAU | 15.5 | LEU | CUA | 9.00 | TRP | UGG | 11.7 |
| ASP | GAC | 25.3 | LEU | CUG | 9.00 | TYR | UAU | 10.4 |
| CYS | UGU | 4.56 | LYS | AAA | 6.70 | TYR | UAC | 17.0 |
| CYS | UGC | 7.47 | LYS | AAG | 15.1 | VAL | GUU | 19.3 |
| GLN | CAA | 14.1 | MET | AUG | 7.82 | VAL | GUC | 12.4 |
| GLN | CAG | 1.56 | PHE | UUU | 8.9 | VAL | GUA | 3.13 |
| GLN | GAA | 18.4 | PHE | UUC | 14.6 | VAL | GUG | 2.87 |
| GLN | GAG | 3.13 | PRO | CCU | 3.13 |  |  |  |
| GLY | GGU | 16.5 | PRO | CCC | 2.01 |  |  |  |
