# Supplementary material for: Ribosome reinitiation can explain length-dependent translation of messenger RNA
Source: PLoS Comput Biol. 2017 Jun 9;13(6):e1005592. doi: 10.1371/journal.pcbi.1005592 (PMC5482490; doi:10.1371/journal.pcbi.1005592)
Supplement: S1 Text — (DOCX) [file pcbi.1005592.s007.docx]

**S1 Text: Parameter Estimates and Justification**

**Transcript lifetime, age distribution and mode of decay**

The decay of eukaryotic transcripts is initiated by the stepwise removal of adenine residues from the poly(A) tail [1]. Deadenylation is the slowest step in mRNA decay, and during this process there is no apparent decay of the transcribed portion of the mRNA [2,3]. Enzymatic digestion of the poly(A) tail is distributive: the deadenylase binds to a poly(A) tail, cleaves a small number of residues, then dissociates from the transcript before binding to a different poly(A) tail [4]. Thus deadenylation consists of a series of sequential first-order reactions, resulting in a hypo-exponential distribution of full-length transcript lifetimes [1,5]. Hypo-exponential distributions, by definition, have lower variances than an exponential function with the same mean lifetime and age-matched populations of eukaryotic transcripts are expected to show very little degradation for long periods then decay rapidly. When the number of sequential deadenylation steps is high (~30), the distribution of transcript lifetimes becomes symmetrical and approximates the normal distribution [6]. We have therefore, for computational simplicity, assumed that all transcripts in a given simulation have the same fixed lifetime.

In our model, all translation ceases as soon as the transcript lifetime is reached; ribosomes that have a new round of translation are not allowed to finish and therefore do not contribute to the protein yield. Consequently, protein yield in our model does not exactly match the effective initiation rate. There is evidence that eukaryotic mRNA decay is co-translational: transcript degradation in the 5'-3' direction follows the last translating ribosome allowing all ribosomes to complete their final round [7]. If this is the sole mechanism of eukaryotic transcript decay, then the true yield becomes the effective initiation rate multiplied by the transcript lifetime (a constant in our model).

***De novo* initiation rates**

The average number of ribosomes per transcript is remarkably constant across eukaryotes. Polysome profiling studies estimate that the median *S. cerevisiae* transcript contains 6.0 ribosomes (based on weighted averages for all measured transcripts [8]; an identical average was calculated in [9]) while the average human embryonic kidney HEK293T cell contains 5.6-6.1 ribosomes [10]. Direct counts of the numbers of ribosomes in polysomes [11] using atomic force microscopy provide similar values (*S. cerevisiae* = 6.5 ribosomes per transcript, HEK293T = 8.7 ribosomes per transcript, human MCF-7 cells = 8.3 ribosomes per transcript). Direct polysome counts are almost certainly over-estimates of the average number of ribosomes per transcript as they ignore unoccupied transcripts (approximately 15-30% of mRNAs [8,10]) and mRNAs occupied by a single ribosome. To be consistent with these counts, in all of our models, for any transcript lifetime and reinitiation probability, we have adjusted the *de novo* initiation rate such that the average transcript (CDS length = 400 codons) carries 6 ribosomes (white line on each heatmap in S2 Figure). This causes the *de novo* initiation rate to decrease with increasing reinitiation level and decreasing transcript lifetimes.

Maintaining six ribosomes on a 400-codon-long transcript for transcripts with different lifetimes requires much larger changes to the *de novo* initiation rate at high reinitiation probabilities compared to low reinitiation levels (S2 Figure). Under perfect reinitiation, the lifetime protein yields of transcripts of a given CDS length will be similar, but the rate of protein production over time (proteins per mRNA per unit time) will be higher for short-lived transcripts compared to long-lived transcripts. Thus, organisms with short-lived transcripts, such as yeast, will exhibit much higher protein synthesis rates than organisms with long-lived transcripts, such as mammals. Current estimates suggest that protein production rates in mammals are considerably lower than in yeast, consistent with the much higher doubling rates and much lower protein stabilities in yeast compared to mammals [12-14]. In contrast, assuming similar elongation rates across species, linear models predict that yeast and mammals should have similar protein production rates since they have similar average ribosome densities.

**Model parameters**

**Full model.** We explored the consequences of different reinitiation levels on the average ribosome density, effective initiation rate and protein yield for transcripts with different CDS lengths using a wide range of transcript lifetimes and *de novo* initiation rates (S2 Figure). For each combination of transcript lifetime and *de novo* initiation rate, we simulated translation for transcripts of the following log-uniform distributed CDS lengths (in codons): 50, 63, 79, 100, 126, 158, 200, 251, 316, 399, 502, 632, 796, 1002, 1263, 1590, 2002, 2522, 3176, and 4000, and then calculated the slope of the resulting estimates of ribosome density, effective initiation rate, and protein yield over CDS length. All codons were decoded at a rate of 10s^-1^ based on the average level in yeast [15] and similar to the average rate observed in a mouse embryonic cell line (5.6s^-1^ [16]). Termination rates (the sum of the release rate and the reinitiation rate) are set equal to the elongation rate at 10s^-1^. Different reinitiation levels are achieved by setting the reinitiation rate as the corresponding proportion of the termination rate. Translation of each transcript was averaged over 1000 runs.

**General model**. To explore the consequences of different reinitiation levels in more detail, we present a model using an arbitrary lifetime of 3000s (50 minutes) for all transcripts (S2-S5 Figures). This value is intermediate between estimates of median transcript half-lives in yeast (10-30 minutes [17]) and mammalian cell lines (300-600 minutes [12,14]). Simulations were performed with a constant elongation rate of 10s^-1^ (except for S3 Figure where we perform the same simulation at 5s^-1^ and 20s^-1^). The model is otherwise the same as the full model, except that a single *de novo* initiation rate is used at each reinitiation level. *De novo* initiation rates are adjusted for each reinitiation level such that a 400-codon-long transcript carries an average of 6 ribosomes. The *de novo* initiation rates used with a transcriptome-wide elongation rate of 10s^-1^ were: 100% = 0.00438s^-1^, 99.9% = 0.00458s^-1^, 99% = 0.00586s^-1^, 95% = 0.01289s^-1^, 90% = 0.02285s^-1^, 80% = 0.04199s^-1^, 50% = 0.09570s^-1^, 0% = 0.17578s^-1^.

**Yeast-specific model**. We computed the average ribosome density, effective initiation rate, and protein yield (Fig. 6) for 5888 *S. cerevisiae* transcripts ranging in CDS length from 16 to 4910 codons (median length = 405 codons) in our model. We used the codon-specific elongation rates calculated by Gilchrist & Wagner [18]; these rates are scaled such that the average elongation rate is 10s^-1^. As above, we adjusted *de novo* initiation rates for each reinitiation level such that a 400-codon-long transcript (ignoring variation in decoding rates) contained an average of 6 ribosomes. The exact *de novo* initiation rates used were: 100% = 0.00859s^-1^, 99.9% = 0.00869s^-1^, 99% = 0.01016s^-1^, 95% = 0.01641s^-1^, 90% = 0.02568s^-1^, 80% = 0.04492s^-1^, 50% = 0.09766s^-1^, 0% = 0.17578s^-1^.

Most studies of mRNA stability report transcript half-lives. If eukaryotic transcripts decay with biphasic (slow-then-fast) kinetics, then transcript lifetimes cannot be calculated from observed half-lives by assuming first-order kinetics [3]. We have therefore based our estimate of transcript lifetime on a study of nascent transcription rates in *S. cerevisiae* which estimated that the entire set of mRNAs in a cell turns over more than four times per 6780s (113 minute) cell cycle [19], resulting in an average transcript lifetime of 1553s (26 minutes). Although most yeast studies predict fairly similar median transcript half-lives, gene-specific estimates show little correlation across studies [17]. Consequently, we have made the simplifying assumption that all transcripts have the same 1553s lifetime.

**Supporting References for S1 Text**

**1.** Cao D, Parker R. Computational modeling of eukaryotic mRNA turnover. RNA. 2001;7: 1192-1212.

**2.** Dreyfus M, Régnier P. The poly(A) tail of mRNAs: bodyguards in eukaryotes, scavenger in bacteria. Cell. 2002;111: 611-613.

**3.** Chen CYA, Ezzeddine N, Shyu AB. Messenger RNA half-life measurements in mammalian cells. Methods Enzymol. 2008;448: 335-357.

**4.** Chen CYA, Shyu AB. Mechanisms of deadenylation-dependent decay. Wiley Interdiscip Rev RNA. 2010;2: 167-183.

**5.** Decker CJ, Parker R. A turnover pathway for both stable and unstable mRNAs in yeast: evidence for a requirement for deadenylation. Genes & Dev. 1993;7: 1632-1643.

**6.** Kuo TC, Huang WC, Wu SC, Cheng PL. A case study of inter-arrival time distributions of container ships. J Mar Sci Techol. 2006;14: 155-164.

**7.** Pelechano V, Wei W, Steinmetz LM. Widespread co-translational RNA decay reveals ribosome dynamics. Cell. 2015;161: 1400-1412.

**8.** Arava Y, Wang Y, Storey JD, Brown PO, Herschlag D. Genome-wide analysis of mRNA translation profiles in *Saccharomyces cerevisiae*. Proc Natl Acad Sci USA. 2003;100: 3889-3894.

**9.** MacKay VL, Li X, Flory MR, Turcott E, Law GL, Serikawa KA, et al. Gene expression analyzed by high-resolution state array analysis and quantitative proteomics. Mol Cell Proteomics. 2004;3: 478-489.

**10.** Hendrickson DG, Hogan DJ, McCullough HL, Myers JW, Herschlag D, Ferrell JE, et al. Concordant regulation of translation and mRNA abundance for hundreds of targets of a human microRNA. PLoS Biol. 2009;7: e1000238.

**11.** Lauria F, Tebaldi T, Lunelli L, Struffi P, Gatto P, Pugliese A, et al. RiboAbacus: a model trained on polyribosome images predicts ribosome density and translational efficiency from mammalian transcriptomes. Nucl Acids Res. 2015;43: e153.

**12.** Jackson DA, Pombo A, Iborra F. The balance sheet for transcription: an analysis of nuclear RNA metabolism in mammalian cells. FASEB J. 2000;14: 242-254.

**13.** von der Haar T. A quantitative estimation of the global translational activity in logarithmically growing yeast cells. BMC Systems Biol. 2008;2: 87.

**14.** Schwanhaüsser B, Busse D, Li N, Dittmar G, Schuchhardt J, Wolf J, et al. Global quantification of mammalian gene expression control. Nature. 2011;473: 337-342.

**15.** Waldron C, Jund R, Lacroute F. The elongation rate of proteins of different molecular weight classes in yeast. FEBS Lett. 1974;46: 11-16.

**16.** Ingolia NT, Lareau LF, Weissman JS. Ribosome profiling of mouse embryonic stem cells reveals the complexity and dynamics of mammalian proteomes. Cell. 2011;147: 789-802.

**17.** Geisberg JV, Moqtaderi Z, Fan X, Ozsolak F, Struhl K. Global analysis of mRNA isoform half-lives reveals stabilizing and destabilizing elements in yeast. Cell. 2014;156: 812-824.

**18.** Gilchrist MA, Wagner A. A model of protein translation including codon bias, nonsense errors, and ribosome recycling. J Theor Biol. 2006;239: 417-434.

**19.** Pelechano V, Chávez S, Pérez-Ortín JE. A complete set of nascent transcription rates for yeast genes. PLoS ONE. 2010;5: e15442.
